# Total Flavonoids from *Berberis kaschgarica* Rupr. Ameliorate Atherosclerosis in ApoE^−/−^ Mice by Regulating Lipid Metabolism and Gut Microbiota

**DOI:** 10.3390/antiox15060703

**Published:** 2026-06-03

**Authors:** Adili Abudureheman, Dilihuma Dilimulati, Yipaerguli Paerhati, Alifeiye Aikebaier, Alhar Baishan, Xiaoxiao Qiu, Nazhakaiti Yusufujiang, Yilixiati Wusiman, Ainiwaer Wumaier, Wenting Zhou

**Affiliations:** 1 Department of Pharmacology, College of Pharmacy, Xinjiang Medical University, Urumqi 830017, China; adil922@xjmu.edu.cn (A.A.);; 2 Xinjiang Key Laboratory of Natural Medicines Active Components and Drug Release Technology, Urumqi 830017, China; 3 Xinjiang Key Laboratory of Biopharmaceuticals and Medical Devices, Urumqi 830017, China; 4 Engineering Research Center of Xinjiang and Central Asian Medicine Resources, Ministry of Education, Urumqi 830017, China

**Keywords:** *Berberis kaschgarica* Rupr., total flavonoids, atherosclerosis, lipid metabolism, gut microbiota, ApoE^−/−^ mice

## Abstract

**Background**: Atherosclerosis is the major pathological basis of cardiovascular diseases, while current lipid-lowering therapies remain limited by suboptimal efficacy and safety. Natural flavonoids, with their multi-target pharmacological activities, are promising candidates for anti-atherosclerotic intervention. This study investigated the protective effects and mechanisms of total flavonoids from *Berberis kaschgarica* Rupr. (BTF) against high-fat diet (HFD)-induced atherosclerosis in ApoE^−/−^ mice. **Methods**: Bioavailable constituents of BTF were identified by UPLC-Q-TOF-MS. Network pharmacology and bioinformatics analyses were performed to predict therapeutic targets and pathways. Male ApoE^−/−^ mice were fed an HFD and treated with low, medium, or high doses of BTF or atorvastatin. Serum lipid profiles, oxidative stress markers, and aortic histopathology were evaluated. Hepatic proteins related to lipid metabolism were measured by Western blotting, and fecal gut microbiota were analyzed by 16S rRNA sequencing. **Results**: Seven flavonoid monomers, including kaempferol, apigenin, calycosin, and dihydromyricetin, were identified as absorbed constituents in serum. Bioinformatics suggested that BTF regulates targets involved in lipid metabolism, oxidative stress, and inflammation. BTF dose-dependently decreased serum TC, TG, LDL-C, MDA, and LDH, while increasing HDL-C and the activities of SOD, GSH, and CAT. BTF also reduced atherosclerotic plaque formation and preserved aortic wall structure. Mechanistically, BTF downregulated hepatic *SREBP1*, *ACC1*, *FAS*, *APOB*, and *MTP* expression and improved HFD-induced gut microbiota dysbiosis by enriching Akkermansia and Lactobacillus. **Conclusions**: BTF attenuated HFD-induced atherosclerosis by improving lipid metabolism, enhancing antioxidant capacity, and modulating gut microbiota composition, supporting its potential as a natural anti-atherosclerotic agent.

## 1. Introduction

Atherosclerosis is a long-term vascular disease and the pathological basis of many major cardiovascular events, including coronary heart disease, ischemic stroke, and peripheral arterial disorders [1]. The formation and progression of atherosclerotic lesions involve a series of interrelated events, such as abnormal lipid accumulation, endothelial injury, chronic inflammatory responses, oxidative damage, and structural changes in the arterial wall [2]. Among these factors, oxidative stress plays a particularly important role in disease progression. Excessive generation of reactive oxygen species can trigger low-density lipoprotein oxidation, damage endothelial cells, intensify inflammatory signaling, and thereby promote plaque development. In addition, disordered hepatic lipid metabolism and disruption of intestinal microbial balance are increasingly recognized as important contributors to atherosclerotic progression. For this reason, therapeutic agents capable of regulating lipid metabolism while simultaneously reducing oxidative injury and inflammatory burden may have considerable value in atherosclerosis management.

At present, lipid-lowering treatment remains the cornerstone of atherosclerosis prevention and control, with statins being the most commonly used drugs in clinical practice. Although these agents are effective in lowering cardiovascular risk, their therapeutic outcomes are still limited by residual risk, differences in patient responsiveness, and the possibility of side effects, including muscle-related symptoms and liver function abnormalities [3]. Because atherosclerosis is driven by multiple pathological mechanisms, interventions aimed at only one target are often insufficient. In recent years, naturally occurring compounds have received increasing attention because of their broad biological activities and relatively diverse mechanisms of action. Flavonoids, an important class of plant-derived polyphenolic compounds, have been reported to exert antioxidant, anti-inflammatory, lipid-lowering, and vascular-protective effects [4]. A growing body of evidence suggests that flavonoid-rich preparations may help delay atherosclerotic progression through improvement of redox status, correction of lipid disturbances, and protection of vascular function.

*Berberis kaschgarica* Rupr., a medicinal plant distributed in Xinjiang, China, has been used in Uyghur traditional medicine for conditions associated with cardiovascular dysfunction. *Berberis kaschgarica* Rupr. is rich in a diverse array of bioactive constituents with potential therapeutic value, including amino acids, proteins, carbohydrates, saponins, phenols, organic acids, flavonoids, coumarins, and volatile oils [5]. Previous studies have shown that the fruit extract of *Berberis kaschgarica* Rupr. possesses cardiovascular protective effects such as concentration-dependent vasodilation [6], anti-inflammatory activity, and antihypertensive activity [7]. Nevertheless, the role of total flavonoids from *Berberis kaschgarica* Rupr. (BTF) in atherosclerosis has not yet been fully defined. In particular, there is still insufficient evidence regarding which flavonoid components are absorbed into the circulation, whether BTF can alleviate oxidative stress and lipid metabolic disorders in vivo, and how it may influence gut microbial composition during atherosclerotic progression.

Emerging evidence supports the concept of a gut–liver–vascular connection in the development of atherosclerosis. Enhanced hepatic lipogenesis and increased very low-density lipoprotein secretion can aggravate hyperlipidemia and accelerate plaque formation, whereas gut microbiota imbalance may further worsen oxidative stress and chronic inflammation. Based on these considerations, we proposed that BTF might exert anti-atherosclerotic effects through integrated regulation of antioxidant defense, hepatic lipid metabolism, and intestinal microbiota homeostasis.

To test this hypothesis, the present study combined UPLC-Q-TOF-MS-based serum component analysis, network pharmacology, an ApoE^−/−^ mouse model of diet-induced atherosclerosis, Western blotting, and 16S rRNA gene sequencing. The objectives were to characterize the absorbed flavonoid constituents of BTF, evaluate its anti-atherosclerotic efficacy in vivo, clarify its effects on oxidative stress and hepatic lipid metabolic pathways, and assess its regulatory influence on gut microbiota. The results of this work may offer experimental support for the development of BTF as a natural intervention for atherosclerosis.

## 2. Materials and Methods

### 2.1. Sample Preparation for UPLC-ESI-MS/MS

The fruits of BKF were collected in Akto County, Kashgar Prefecture, Xinjiang Uygur Autonomous Region, China, in October 2024. The plant material was botanically identified by Prof. Palida Abulizi (Department of Natural Medicinal Chemistry and Pharmacognosy, School of Pharmacy, Xinjiang Medical University). A voucher specimen (No. XJMUYXY20201020) has been stored in the Ethnical Herbs Specimen Museum of Traditional Chinese Medicines at the same university. The freeze-dried BKF samples were crushed at 30 Hz for 1.5 min using a mixer equipped with zirconia beads. Before extraction, rutin (spiked internal loading control, final concentration: 1 μg/mL) was added to all serum samples (blank rat serum and BTF-containing serum) to ensure the accuracy of LC-MS/MS qualitative and quantitative analysis. A quantity of 100 mg of the resulting powder was weighed and extracted with 1.2 mL of a 70% methanol aqueous solution at 4 °C overnight. Following centrifugation at 10,000× *g* for 10 min, the extracts were adsorbed onto a CNWBOND carbon-GCB solid-phase extraction column (ANPEL, Shanghai, China). The samples were then filtered using an SCAA-104 filter (ANPEL, Shanghai, China) and subsequently analyzed employing an ultra-performance liquid chromatography–electrospray ionization–tandem mass spectrometry (UPLC-ESI-MS/MS) system, specifically the Shim-pack UPLC SHIMADZU CBM30A (Shimadzu Corporation, Kyoto, Japan) for UPLC and the Applied Biosystems 6500 Q TRAP (Biosystems, Foster City, CA, USA) for MS/MS analysis.

### 2.2. UPLC Conditions

The analytical conditions for the UPLC method were as follows: the mobile phase consisted of solvent A (pure water with 0.04% acetic acid) and solvent B (acetonitrile with 0.04% acetic acid). Sample measurements were carried out using a gradient program, initially set to 95% A and 5% B. After 10 min, the gradient was linearly adjusted to 5% A and 95% B, which was maintained for 1 min. Subsequently, the composition was rapidly adjusted to 95% A and 5% B within 0.10 min and kept for 2.9 min. The column oven temperature was set at 40 °C, and the injection volume was 2 L. The effluent from the column was connected to an electrospray–triple quadrupole–linear ion trap (Q TRAP)-MS instrument.

### 2.3. ESI-Q TRAP-MS/MS

The linear ion trap and triple quadrupole (QQQ) scans were obtained using a Q TRAP-MS system, specifically the API 6500 Q TRAP UPLC/MS/MS system. The system is outfitted with an ESI Turbo ion spray interface, capable of operating in both positive and negative ionization modes. Control of the system is facilitated by Analyst 1.7.2 software (SCIEX, Singapore). The operational parameters for the ESI source are specified as follows: the ion source employed is a turbo spray, with a source temperature maintained at 550 °C. The ion spray voltage is configured to 5500 V for the positive ion mode and −4500 V for the negative ion mode. The ion source gases I and II, along with the curtain gas, are set at pressures of 50 psi, 60 psi, and 30 psi, respectively. Additionally, the collision gas was set to a high level. To ensure accurate measurement, the instrument was tuned and quality calibrated using 10 and 100 mol/L polypropylene glycol solutions in QQQ and linear ion trap modes, respectively. During the QQQ scan, a collision gas (nitrogen) pressure of 5 pounds per square inch was used for the multiple reaction monitor (MRM) experiment. Individual MRM transitions were optimized based on the declustering potential and collision energy, and further optimization of the declustering potential and collision energy was performed. Based on the eluted metabolites, a specific set of MRM transitions was monitored for each period.

### 2.4. Network Pharmacological Analysis

Standardized molecular structures of BTF were obtained as SMILES (Simplified Molecular-Input Line-Entry System) identifiers, sourced from the PubChem database (http://pubchem.ncbi.nlm.nih.gov (accessed on 5 March 2026)). (The targets of BTF on humans were obtained from databases such as Traditional Chinese Medicine Systems Pharmacology (TCMSP) (https://www.tcmsp-e.com (accessed on 7 March 2026)), PharmMapper (https://lilab-ecust.cn/pharmmapper/ (accessed on 7 March 2026)), ChemMapper, and SwissTargetPrediction (http://swisstargetprediction.ch (accessed on 7 March 2026)). Human-related targets of AS were collected using DisGeNET (http://www.disgenet.org), OMIM (http://www.omim.org), Gene Expression Omnibus (GEO) database (https://www.ncbi.nlm.nih.gov/geo), and GeneCards databases (http://www.genecards.org (accessed on 9 March 2026)). Subsequently, DAVID bioinformatics resources (https://davidbioinformatics.nih.gov (accessed on 9 March 2026)) were utilized to perform GO functional enrichment analysis and KEGG pathway analysis on the interaction targets. Cytoscape 3.6.1 (http://www.cytoscape.org) was utilized for visualizing the network of drug-disease interaction targets.

### 2.5. Preparation of BTF

The dried plant material of *Berberis kaschgarica* Rupr. was pulverized and passed through a 40-mesh sieve. An appropriate amount of powder was extracted twice with 70% ethanol at a solid–liquid ratio of 1:10 (*w*/*v*) under reflux, with each extraction performed for 1.5 h. The extracts were combined, filtered, and concentrated under reduced pressure at 45 °C to remove the solvent and obtain a crude extract.

The crude extract was then resuspended in distilled water and purified using a macroporous adsorption resin column. After sample loading, the column was washed with distilled water to remove polar impurities, including sugars and small water-soluble components. Subsequently, the flavonoid-rich fraction was eluted with 70% ethanol. The eluate was collected, concentrated under vacuum, and freeze-dried to yield the total flavonoids of *Berberis kaschgarica* Rupr. (BTF). The final extract was stored at 4 °C in a dry, light-protected environment until further use.

The total flavonoid content of BTF was determined by aluminum chloride colorimetry with rutin as the reference compound, and the content was expressed as rutin equivalents.

### 2.6. Zebrafish Culture and Basic Indicator Testing

Adult AB wild-type zebrafish at three months of age were obtained from the Wuhan Institute of Aquatic Biology, Chinese Academy of Sciences. Male and female fish were placed in transparent glass aquariums in equal numbers, with a partition set inside for isolation. Partitions were taken away early in the morning on the next day to allow natural mating and spawning. Embryos were incubated at 28 °C for five days. Well-developed larval fish were screened under a stereomicroscope and randomly divided into experimental groups. Animals were fed with a 5% high-cholesterol diet (Xiaoshuyoutai Biotechnology Co., Ltd., Beijing, China) and treated with different doses of total flavonoids. General physiological indices of zebrafish in each group were measured after 10 days of continuous feeding.

### 2.7. Animals and Treatments

Male ApoE^−/−^ mice and age-matched wild-type mice were obtained from the Tongxiang Branch, Zhejiang, China, and maintained under standard laboratory conditions with free access to food and water. After acclimatization, male ApoE^−/−^ mice (1 week old) were randomly divided into six groups (*n* = 6): (1) Control group (C57BL/6J mice, standard chow); (2) Model group (ApoE^−/−^, HFD); (3) BTF-L (low-dose BTF, 20 mg/kg); (4) BTF-M (medium-dose BTF, 40 mg/kg); (5) BTF-H (high-dose BTF, 60 mg/kg); (6) Atorvastatin group (10 mg/kg). All treatments were administered daily by oral gavage for 16 consecutive weeks. Body weight was monitored weekly. At the end of the experiment, blood, liver, thoracic aorta, and fecal samples were harvested for further analysis. All animal experiments were conducted in strict compliance with the ARRIVE Guidelines 2.0 and the Guide for the Care and Use of Laboratory Animals. The experimental protocol was approved by the Xinjiang Medical University Experimental Animal Ethics Committee (Ethical Approval Code: IACUC-JT-20250911-51).

### 2.8. ELISA

Serum biochemical indices, including total cholesterol (TC) (Nanjing Jiancheng Bioengineering Institute, Nanjing, China; Lot No. A111-2), triglycerides (TG) (Nanjing Jiancheng Bioengineering Institute, Nanjing, China; Lot No. A110-2-1), LDL-cholesterol (LDL-C) (Nanjing Jiancheng Bioengineering Institute, Nanjing, China; Lot No. A113-1), and HDL-cholesterol (HDL-C) (Nanjing Jiancheng Bioengineering Institute, Nanjing, China; Lot No. A112-1), were quantified via standard enzymatic colorimetric assays. We measured malondialdehyde (MDA) (Nanjing Jiancheng Bioengineering Institute, Nanjing, China; Lot No. A003-1-2), lactate dehydrogenase (LDH) (Nanjing Jiancheng Bioengineering Institute, Nanjing, China; Lot No. A020-2-2), superoxide dismutase (SOD) (Nanjing Jiancheng Bioengineering Institute, Nanjing, China; Lot No. A001-3-2) glutathione (GSH) (Nanjing Jiancheng Bioengineering Institute, Nanjing, China; Lot No. A006-2-1), and catalase (CAT) (Nanjing Jiancheng Bioengineering Institute, Nanjing, China; Lot No. A007-1-1) via commercial kits, following standard manufacturer guidelines.

### 2.9. Histopathological Examination of Aorta

Thoracic aortas were fixed, dehydrated, embedded in paraffin, and sectioned at 5 μm thickness. HE staining was used to assess morphological changes, intimal thickening, plaque formation, and tissue integrity. Light microscopic images were captured and analyzed by blinded observers.

### 2.10. Western Blot Analysis

Liver tissue samples were lysed in RIPA buffer (Servicebio, Wuhan, China; Lot No. G2002) to extract total intracellular protein. The BCA assay was then applied to calibrate the overall protein concentration. Prepared protein samples with consistent loading quantities were separated through SDS-PAGE electrophoresis, followed by transmembrane transfer onto PVDF membranes. After the blocking step, all membranes were incubated overnight at 4 C with primary antibodies targeting *SREBP1* (BIOSS, Beijing, China; Lot No. bs-1402R), *ACC1* (BIOSS, Beijing, China; Lot No. bsm-61228R), *FAS* (BIOWORLD, St. Louis Park, MN, USA; Lot No. BS40387), *ApoB* (BIOWORLD, St. Louis Park, MN, USA; Lot No. BS70165), *MTP* (BIOWORLD, St. Louis Park, MN, USA; Lot No. BS6672), and *GAPDH* (Xianzhi Biological, Fuzhou, China; Lot No. AB-P-R 001). Following the washing procedures, the blots were treated with the appropriate secondary antibodies. An enhanced chemiluminescence system was employed to visualize the target protein bands, and the gray intensity of the bands was subsequently measured to conduct a quantitative analysis.

### 2.11. Gut Microbiota Analysis

Fresh fecal specimens were collected from experimental subjects and processed to isolate microbial genomic DNA. Amplification was targeted at the V3–V4 segment of the 16S rRNA gene, with high-throughput sequencing completed on the Illumina sequencing system (Illumina MiSeq PE300, San Diego, CA, USA). Related bioinformatics workflows were applied to evaluate microbial alpha and beta diversity, bacterial community structure, and intergroup species discrepancies. LEfSe and LDA score analysis were applied to identify key phylotypes responsible for group differences.

### 2.12. Statistical Analysis

All data are expressed as mean ± standard deviation (SD). Statistical comparisons were performed using one-way analysis of variance (ANOVA) followed by Tukey’s post hoc test. A *p*-value < 0.05 was considered statistically significant.

## 3. Results

### 3.1. Identification of Blood-Exposed Constituents of BTF by UPLC–MS/MS

**Figure 1:**
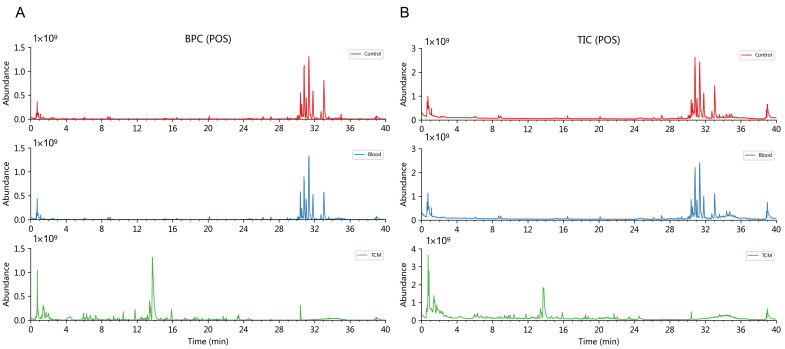
UPLC-MS/MS chromatograms of BTF in rat serum: (**A**) Base peak chromatograms (BPCs) of blank serum (Control), drug-containing serum (blood), and BTF extract (TCM) in positive ion mode. (**B**) Total ion chromatograms (TICs) of blank serum, drug-containing serum, and BTF extract in positive ion mode.

To define the pharmacodynamic material basis of BTF, we analyzed rat serum after oral administration using UPLC-MS/MS in both positive and negative ion modes. Representative base peak chromatograms (BPCs) and total ion chromatograms (TICs) of blank serum, drug-containing serum, and BTF extract are shown in Figure 1A,B.

The chromatographic profiles of blank serum (red trace) showed only endogenous peaks, with no signals corresponding to flavonoids. In contrast, drug-containing serum (blue trace) exhibited multiple distinct peaks at retention times of 20–34 min, which were absent in controls and matched the elution pattern of the parent BTF extract (green trace). These findings confirmed that flavonoid constituents from BTF were absorbed into the systemic circulation.

To ensure the reliability of compound identification, rutin was spiked as an internal loading control into all serum samples before UPLC-Q-TOF-MS analysis. Seven major absorbable flavonoid compounds were identified by accurate mass, isotopic distribution, and MS/MS fragmentation data (Table 1). These included kaempferol, diosmetin, apigenin, acacetin, fortunellin, dihydromyricetin, and calycosin. All of these compounds have been reported to possess lipid-regulating, antioxidant, or anti-inflammatory activities [8,9,10,11], supporting their potential contribution to the observed in vivo effects of BTF.

### 3.2. Network Pharmacology Analysis Identified Potential Anti-Atherosclerotic Targets and Pathways of BTF

**Figure 2:**
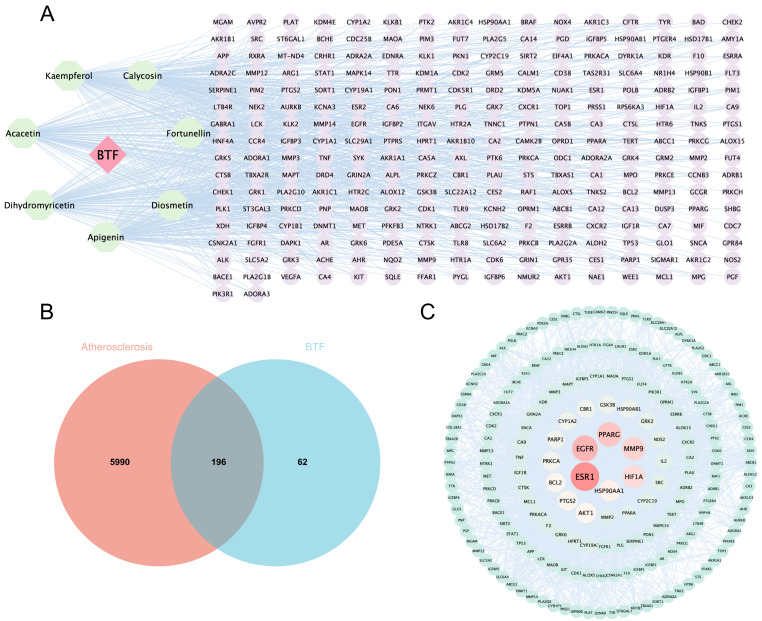
Network pharmacology analysis of BTF against atherosclerosis: (**A**) Compound–target interaction network of the seven absorbed flavonoid components of BTF. The pink diamond represents BTF; green hexagons represent the identified flavonoid compounds; purple circles represent the predicted targets. Edges indicate compound–target interactions. (**B**) Venn diagram showing the intersection between BTF-related targets and atherosclerosis-related genes. A total of 196 overlapping genes were identified. (**C**) Protein–protein interaction (PPI) network of the 196 overlapping targets. Hub genes including ESR1, HSP90AA1, EGFR, MMP9, PPARG, and AKT1 are highlighted.

To explore the multi-target mechanisms of BTF, we first constructed a compound–target interaction network based on the seven absorbed flavonoid constituents (Figure 2A). The network revealed that BTF acts on a broad spectrum of targets closely associated with vascular homeostasis and lipid metabolism.

**Figure 3:**
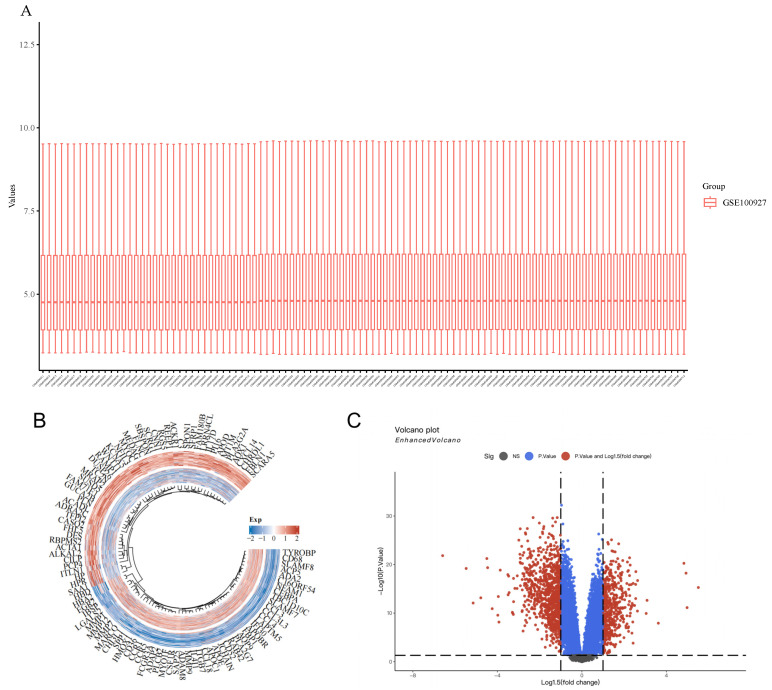
Differential gene expression analysis based on the GSE100927 dataset: (**A**) Bar plot showing the expression levels of representative genes across samples in the GSE100927 dataset. (**B**) Heatmap of the top differentially expressed genes (DEGs) in atherosclerosis. Red and blue indicate upregulated and downregulated expression, respectively. (**C**) Volcano plot of DEGs in the atherosclerosis dataset. Red points indicate significantly dysregulated genes (*p* < 0.05 and |log_2_FC| > 1); blue points indicate genes with only *p* < 0.05; gray points indicate non-significant genes.

**Figure 4:**
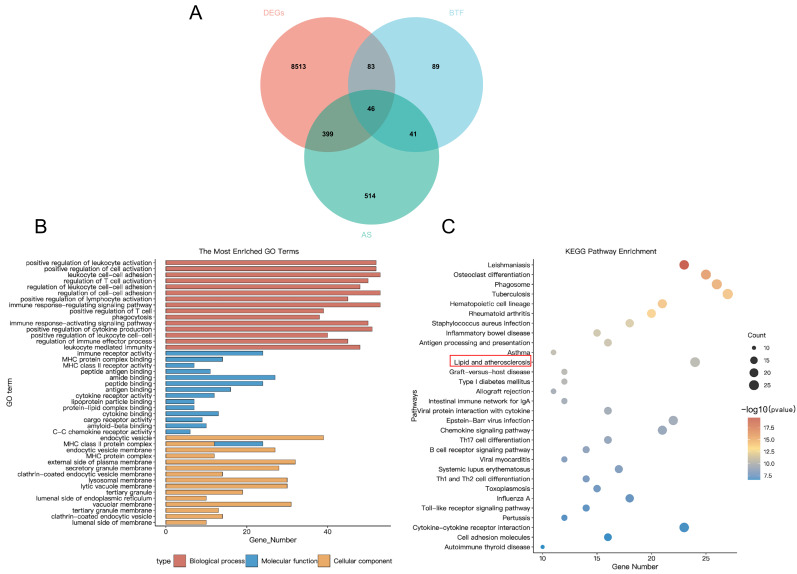
Integrated bioinformatic analysis of key therapeutic targets: (**A**) Three-way Venn diagram showing the intersection of BTF-related targets, atherosclerosis-related genes, and DEGs from the GSE100927 dataset. A total of 46 core hub genes were identified. (**B**) GO enrichment analysis of the 46 core hub genes. The top enriched terms in biological process, molecular function, and cellular component are shown. (**C**) KEGG pathway enrichment analysis of the core hub genes. The size of each dot represents the number of genes enriched in the pathway, and the color gradient indicates the significance (−log_10_ *p*-value). “Lipid and atherosclerosis” was the most significantly enriched pathway.

To identify core therapeutic targets, we intersected BTF-related targets with atherosclerosis-related genes. A total of 196 overlapping genes were obtained (Figure 2B), which were further filtered against differentially expressed genes (DEGs) from the GSE100927 dataset. The volcano plot (Figure 3C) confirmed significant transcriptional dysregulation of atherosclerosis-related genes in the disease model. We then performed a three-way Venn analysis among BTF targets, atherosclerosis-related genes, and DEGs, identifying 46 key hub genes at the intersection (Figure 4A).

Protein–protein interaction (PPI) network analysis of these hub genes revealed a highly interconnected module centered on *ESR1*, *HSP90AA1*, *EGFR*, *MMP9*, *PPARG*, and *AKT1* (Figure 2C), all of which are known regulators of lipid metabolism, inflammation, and plaque stability.

Functional enrichment showed that these core targets were prominently enriched in signaling pathways involved in leukocyte activation, immune regulation, and cellular lipid metabolism (Figure 4B). Advanced KEGG pathway enrichment analysis further pinpointed the “Lipid and Atherosclerosis” cascade as the top-enriched signaling route (Figure 4C), in addition to multiple immune and inflammatory signaling axes. Collectively, these data demonstrate that BTF can attenuate atherosclerotic lesions largely by maintaining lipid balance and restraining excessive inflammatory reactions.

### 3.3. BTF Reduces Lipid Accumulation in a Zebrafish Model of Hyperlipidemia

**Figure 5:**
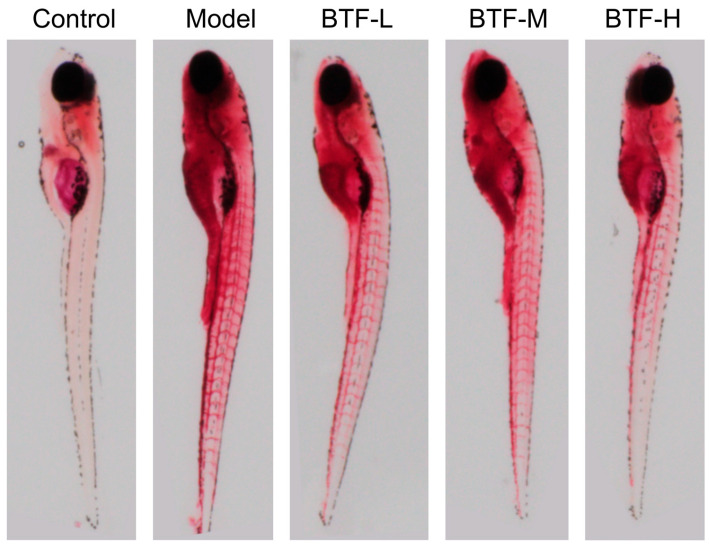
Oil Red O staining for lipid deposition analysis in high-cholesterol zebrafish with different treatments (Original magnification: 5×).

To further evaluate the in vivo lipid-modulating capacity of BTF, Oil Red O staining was conducted using a diet-induced hyperlipidemic zebrafish model. Representative whole-mount staining photographs are presented in Figure 5.

The control group showed minimal neutral lipid deposition, with faint staining confined to visceral tissues. In contrast, the hyperlipidemic model group exhibited widespread oil red O staining, indicating marked systemic lipid accumulation, mainly in the liver and vascular tissues. BTF intervention lowered lipid staining intensity in a dose-dependent fashion. The high-dose BTF group (BTF-H) achieved notable mitigation of aberrant lipid deposition, with staining profiles closely resembling those of healthy control subjects.

Together, these results show that BTF alleviates excessive lipid deposition in vivo and provides direct visual evidence for its lipid-regulating and anti-atherosclerotic effects.

### 3.4. BTF Ameliorates Dyslipidemia, Attenuates Aortic Atherosclerotic Lesions, and Mitigates Oxidative Stress in ApoE^−/−^ Mice

**Figure 6:**
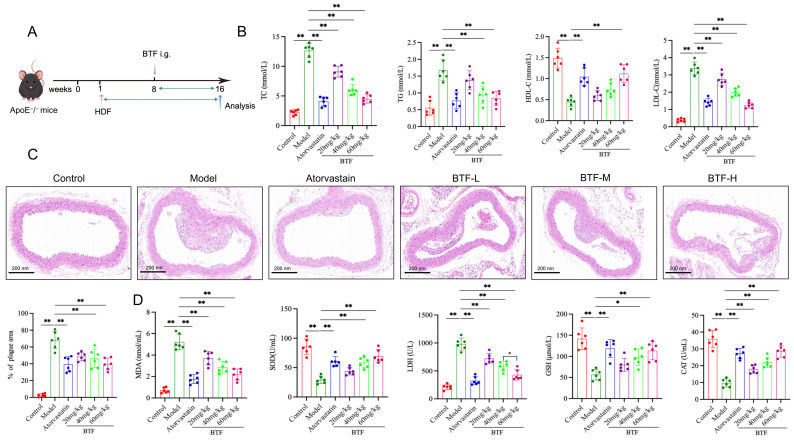
BTF intervention ameliorates serum lipid metabolism, limits atherosclerotic lesion formation, and mitigates oxidative stress in high-fat diet (HFD)-fed mice: (**A**) Schematic diagram of the in vivo experimental design. ApoE^−/−^ Mice were given a continuous HFD beginning at week 1 to establish atherosclerotic injury. From week 8 to week 16, animals received daily intragastric treatment with low, medium, and high doses of BTF (20, 40, and 60 mg/kg/day, defined as BTF-L, BTF-M, and BTF-H); atorvastatin as the positive control (10 mg/kg/day); or a blank vehicle. All animals were euthanized at week 16 for subsequent pathological and biochemical detection. (**B**) Quantification of serum lipid parameters across groups: total cholesterol (TC), triglycerides (TG), high-density lipoprotein cholesterol (HDL-C), and low-density lipoprotein cholesterol (LDL-C). (**C**) Representative hematoxylin and eosin (H&E)-stained cross-sections of the aortic root. Scale bar: 200 nm. Atherosclerotic plaque area was markedly reduced in BTF-treated mice compared with the model group. (**D**) Serum levels of oxidative stress-related biomarkers: malondialdehyde (MDA), superoxide dismutase (SOD), lactate dehydrogenase (LDH), reduced glutathione (GSH), and catalase (CAT). All data are presented as mean ± standard deviation (SD). * *p* < 0.05; ** *p* < 0.01.

To further verify the anti-atherosclerotic effects of BTF in vivo, we used ApoE^−/−^ mice fed a high-fat diet as a preclinical model (Figure 6A). After 16 weeks of treatment, serum lipid levels were measured. As illustrated in Figure 6B,C, the model group demonstrated pronounced dyslipidemia, evidenced by a marked increase in total cholesterol (TC), triglycerides (TG), and low-density lipoprotein cholesterol (LDL-C), accompanied by a decrease in high-density lipoprotein cholesterol (HDL-C). BTF ameliorated serum lipid profiles in a dose-dependent manner. High-dose BTF downregulated atherogenic lipid markers (TC, TG, and LDL-C) and elevated HDL-C levels, with effects comparable to the positive drug atorvastatin. As shown in Figure 6D, compared with the HFD model group, BTF treatment markedly reduced the contents of MDA and LDH, while significantly elevating the enzymatic activities of SOD, GSH and CAT in mice.

### 3.5. BTF Suppresses Hepatic Lipid Metabolism via the SREBP1 Signaling Pathway

**Figure 7:**
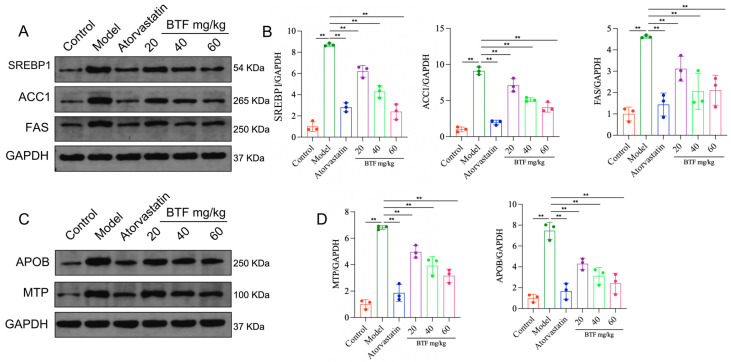
Effects of BTF on the expression of key lipid metabolism-regulating proteins in atherosclerotic ApoE^−/−^ mice: (**A**) Representative Western blot bands showing the expression of lipogenic proteins (*SREBP1*, *ACC1*, and *FAS*) in mouse liver tissues. *GAPDH* was used as the internal loading control. (**B**) Densitometric quantification of the relative protein levels of *SREBP1*, *ACC1*, and *FAS*, normalized to *GAPDH*. (**C**) Representative Western blot images of lipid transport-related proteins (*APOB* and *MTP*) in liver tissues, with *GAPDH* as the internal control. (**D**) Quantitative analysis of *APOB* and *MTP* protein expression, normalized to GAPDH. ** represents *p* < 0.01.

To clarify the molecular mechanism mediating BTF’s lipid-lowering action, we detected the expression of core proteins associated with hepatic lipogenesis and lipoprotein assembly in the liver tissues of ApoE^−/−^ mice (Figure 7A,C).

Western blot results indicated that sustained high-fat diet exposure elevated the protein expression of mature *SREBP1*, downstream lipogenic enzymes *ACC1*, and *FAS* (Figure 7B). These proteins serve as core modulators governing endogenous fatty acid synthesis. BTF intervention downregulated the expression of the three key factors in a dose-related manner; obvious declines in mature *SREBP1* and its downstream targets were observed in the high-dose cohort.

We further investigated the expression of *APOB* and *MTP*, which are essential for hepatic VLDL assembly and secretion (Figure 7D). Both proteins were significantly elevated in the model group, consistent with increased production of atherogenic lipoproteins. BTF treatment significantly downregulated *APOB* and *MTP* expression in a dose-dependent manner, indicating that BTF also inhibits hepatic lipoprotein secretion.

These results confirm that BTF exerts its lipid-regulating effects by inhibiting the *SREBP1*-mediated lipogenic program, thereby reducing both de novo fatty acid synthesis and VLDL secretion in the liver.

### 3.6. BTF Modulates Gut Microbiota Composition in ApoE^−/−^ Mice

To investigate whether BTF’s anti-atherosclerotic effects are associated with gut microbiota remodeling, we performed 16S rRNA gene sequencing on fecal samples from control, model, and BTF-treated mice.

**Figure 8:**
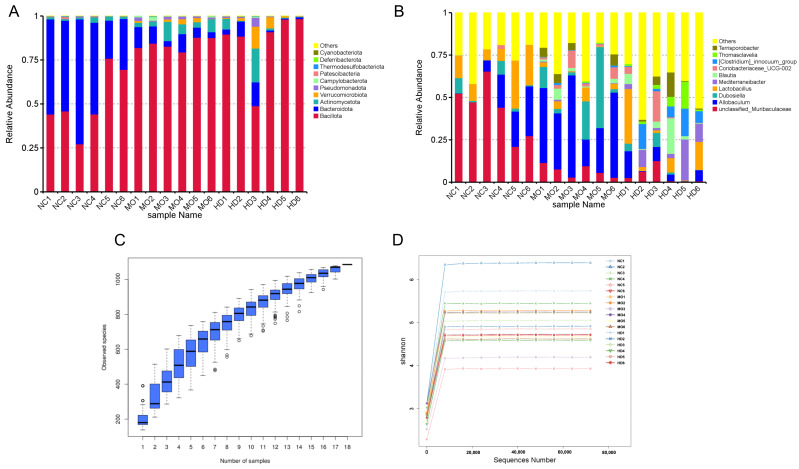
Gut microbial composition and diversity in the NC (normal control), MO (atherosclerotic model), and HD (high-dose BTF) groups: (**A**) Stacked bar charts of bacterial phyla per sample. (**B**) Relative abundance of the top 15 genera. Each bar is one sample; colors denote genera (see legend). (**C**) Species accumulation curve (observed species index). The curve flattens as the number of samples increases, indicating that our sample size captured most gut microbial taxa. (**D**) Rarefaction curves (Shannon index). All curves reach a plateau with increasing sequencing depth, confirming that the depth sufficiently captured the microbial diversity in each sample.

First, we examined the alpha diversity of the gut microbiota. Rarefaction curves (Figure 8C) and Shannon index evaluations (Figure 8D) confirmed that our sequencing efforts achieved adequate depth to encompass the majority of microbial species. The model group demonstrated reduced microbial diversity relative to the control mice, whereas the administration of BTF effectively mitigated this adverse alteration. At the phylum level, the model group demonstrated an altered relative abundance of major bacterial taxa, characterized by a notable increase in Bacillota and a decrease in Bacteroidota (Figure 8A). The administration of BTF treatment effectively reversed this imbalance in a dose-dependent manner, enhancing the proportion of Bacteroidota while reducing Bacillota. At the genus level, the model group showed a significant enrichment of Dubosiella and a reduction in Lactobacillus (Figure 7B). BTF treatment significantly augmented the relative abundance of beneficial genera, such as Lactobacillus and Allobaculum, while decreasing pro-inflammatory taxa.

**Figure 9:**
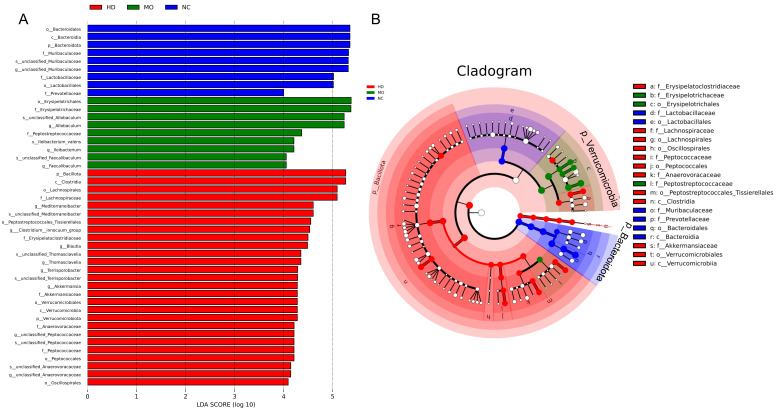
LEfSe analysis identified gut microbial biomarkers linked to atherosclerosis progression and BTF intervention: (**A**) Linear discriminant analysis (LDA) score distribution. We ranked bacterial taxa with significantly different abundances between groups by their LDA scores (log10 scale, threshold > 4.0, *p* < 0.05). Bar length reflects the effect size. Colors indicate the group where each taxon is most abundant: blue, NC (normal control); green, MO (atherosclerosis model); red, HD (high-dose BTF). Annotations show taxonomic levels: p_ (phylum), c_ (class), o_ (order), f_ (family), g_ (genus), and s_ (species). (**B**) Cladogram of differentially abundant taxa. Circles expanding from the center outwards represent taxonomic levels, from phylum to species. Node size corresponds to relative abundance. Colored nodes mark taxa enriched in NC (blue), MO (green), or HD (red). The legend provides detailed taxonomic information for each node.

To identify key bacterial taxa associated with BTF intervention, we performed LEfSe analysis (Figure 9A,B). The cladogram and LDA score plot revealed distinct microbial biomarkers across groups. In the control group, taxa associated with Bacteroidota were predominant, whereas in the atherosclerotic model mice, there was an expansion of Verrucomicrobiota and Erysipelotrichaceae. Supplementation with BTF resulted in an increased abundance of Lactobacillaceae and Muribaculaceae, two beneficial bacterial families known to support the integrity of the intestinal barrier and maintain metabolic homeostasis.

## 4. Discussion

### 4.1. Overview of the Present Study

**Figure 10:**
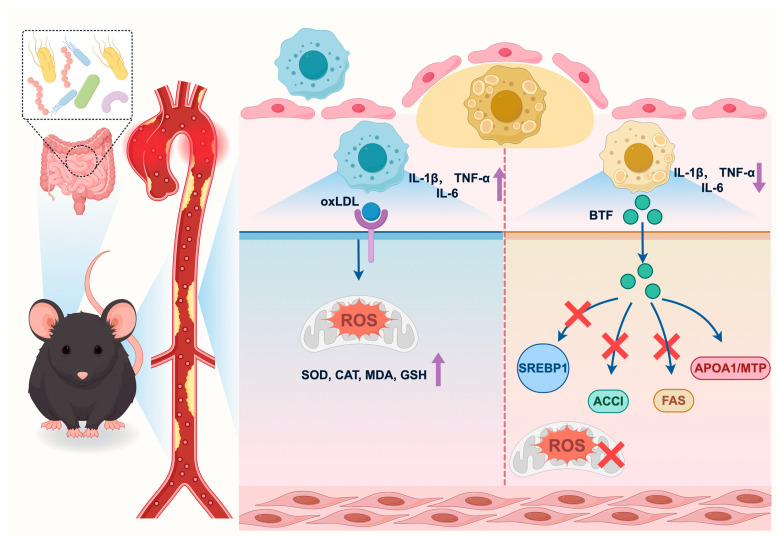
Multi-target anti-atherosclerotic mechanisms of BTF in ApoE^−/−^ mice (schematic diagram). Created in Figdraw 2.0. Zou, W. (2026) (https://www.figdraw.com/, accessed on 27 April 2026).

Atherosclerosis acts as the primary driver of myocardial infarction, ischemic stroke, and peripheral arterial disease and represents a severe long-term health threat worldwide [2]. Statins and other lipid-lowering agents are widely prescribed, yet multiple clinical limitations still exist: residual cardiovascular risk, myalgia, hepatic dysfunction, and insufficient therapeutic response in certain patient populations [12]. Natural flavonoids are attracting growing attention as multi-target, well-tolerated candidates for atherosclerosis intervention [13]. In the present study, we explored the anti-atherosclerotic effects and mechanisms of total flavonoids derived from *Berberis kaschgarica* Rupr. (BTF), a traditional Uyghur medicinal herb. Using UPLC-Q-TOF-MS, we identified seven major flavonoid components absorbed into the systemic circulation after oral administration. Network pharmacology and bioinformatics analysis indicated that these flavonoids regulate pathways involved in lipid metabolism, oxidative stress, and inflammation, with *SREBP1* functioning as a key regulatory factor. In high-fat diet-fed ApoE^−/−^ mice, BTF treatment dose-dependently improved dyslipidemia, reduced aortic plaque formation, alleviated systemic oxidative stress, and suppressed hepatic *SREBP1*-dependent lipogenesis and VLDL secretion. 16S rRNA gene sequencing results demonstrated that BTF alleviated HFD-induced gut dysbiosis and enhanced the abundance of beneficial commensal bacteria. Together, these results indicate that BTF alleviates atherosclerosis by regulating hepatic lipid metabolism and gut microbiota—two key pathways that reduce lesions and improve metabolic health. This supports further development of BTF as a multi-target natural anti-atherosclerotic agent (Figure 10).

### 4.2. The Absorbed Flavonoid Composition: The Pharmacodynamic Basis of BTF

To clarify the underlying mechanism of BTF, we first sought to identify which of its components are absorbed and bioavailable in the body. Using UPLC-Q-TOF-MS, we detected seven major flavonoids in the serum of rats following oral administration of BTF: kaempferol, apigenin, diosmetin, acacetin, calycosin, dihydromyricetin, and fortunellin. Numerous studies have independently documented that these compounds exhibit lipid-lowering, antioxidant, anti-inflammatory, or endothelial-protective properties. This evidence supports the hypothesis that BTF operates as a multi-component synergistic system, rather than depending on a single active constituent.

Kaempferol is one of the most abundant aglycones in BTF. Previous studies have shown that it inhibits *SREBP1* maturation and de novo lipogenesis. It also suppresses foam cell formation and increases antioxidant enzyme activity [8]. Apigenin and diosmetin share structural similarities. Emerging evidence indicates that these two flavonoids suppress *ACC1* and *FAS*, lessen hepatic fat buildup, and safeguard vascular function [9]. Dihydromyricetin has strong free radical-scavenging activity and inhibits vascular inflammation [10]. Calycosin, a typical isoflavone, ameliorates lipid profiles and preserves vascular integrity. Collectively, these bioactive components exert complementary effects on lipid synthesis, oxidative stress, and vascular homeostasis [11]. Identifying these absorbed bioactive compounds clarifies the material foundation of BTF. Unlike most herbal investigations that merely analyze in vitro ingredients without verifying in vivo absorption, our study confirms the truly absorbable components.

### 4.3. BTF Ameliorates Atherosclerosis by Improving Dyslipidemia and Reducing Aortic Plaque Burden

BTF exerted prominent therapeutic effects in HFD-fed ApoE^−/−^ mice, which aligned well with our in silico prediction outcomes. Consistent with experimental expectations, long-term high-fat feeding triggered severe hyperlipidemia: it elevated serum TC, TG, and LDL-C levels, alongside a notable reduction in HDL-C content. BTF treatment significantly and dose-dependently reversed these changes, with high-dose BTF showing lipid-lowering efficacy comparable to atorvastatin. Oil Red O staining showed that BTF reduced atherosclerotic lesions, preserved vascular structure, and limited necrotic core enlargement. These findings demonstrate that BTF slows atherosclerosis. This effect was observed in a well-established mouse model.

BTF showed strong anti-atherosclerotic effects without causing obvious adverse effects. This favorable safety profile indicates that BTF may serve as a well-tolerated, auxiliary therapy for atherosclerosis. Statins reduce cholesterol levels through the inhibition of endogenous synthesis. By comparison, BTF exerts broad regulation over multiple lipid biomarkers simultaneously, including triglycerides and HDL-C. Both indices are well-recognized independent risk factors for cardiovascular disorders. This comprehensive lipid-regulating profile offers distinct advantages for long-term cardiovascular protection, particularly among high-risk individuals.

### 4.4. BTF Inhibits Hepatic Lipogenesis via the SREBP1-Dependent Pathway

Hepatic lipid metabolism is a central determinant of systemic lipid homeostasis and atherosclerotic susceptibility. The transcription factor *SREBP1* turns on lipogenic genes like *ACC1* and *FAS*, which then boost fatty acid and triglyceride synthesis [14]. In hyperlipidemic states, *SREBP1* becomes overactive, boosting fat production in the liver and increasing the release of atherogenic lipoproteins [15]. HFD increased mature *SREBP1*, *ACC1*, and *FAS* protein levels, while BTF reduced these lipogenic proteins in a dose-dependent manner [16]. These findings suggest that BTF suppresses hepatic lipogenesis, thereby limiting the excess lipid accumulation that drives atherosclerosis.

We further examined *APOB* and *MTP*—two critical proteins involved in VLDL assembly. BTF notably downregulated the expression of these two proteins, which in turn suggests a reduction in VLDL biosynthesis. BTF inhibited *APOB* and *MTP*, two essential mediators governing VLDL formation, thereby lowering the generation of atherogenic lipoproteins. This combined mechanism, which blunts lipogenesis and cuts down VLDL secretion, improves abnormal lipid metabolism and accounts for BTF’s potent anti-atherosclerotic capacity.

This study provides the first evidence that BTF alleviates atherosclerosis through direct regulation of the *SREBP1*–*ACC1*–*FAS*–*APOB*–*MTP* axis. These findings provide a clear molecular mechanism linking BTF to lipid metabolism regulation and strongly support the predictions from network pharmacology.

### 4.5. BTF Alleviates Systemic Oxidative Stress and Vascular Injury

Oxidative stress accelerates atherosclerotic progression via endothelial injury, alongside excessive LDL oxidation and sustained inflammatory responses [17]. HFD-fed mice displayed elevated MDA and LDH levels, along with suppressed activities of SOD, GSH, and CAT. These changes reflected pronounced oxidative stress and tissue injury [18]. BTF intervention effectively reversed these abnormal biochemical parameters, thereby boosting endogenous antioxidant defense and mitigating excessive lipid peroxidation [19].

The antioxidant effects of BTF are likely mediated by its flavonoid components, which function as free-radical scavengers and upregulate endogenous antioxidant defense pathways. By reducing oxidative stress, BTF not only lowers LDL oxidation but also preserves endothelial integrity, thereby limiting the initiation and progression of atherogenesis. This antioxidant activity complements its lipid-lowering effects, forming a multidimensional protective system against atherosclerosis.

### 4.6. BTF Restores Gut Microbiota Homeostasis in Atherosclerotic Mice

Emerging evidence has firmly established gut microbiota dysbiosis as an independent contributor to atherosclerosis [3]. HFD feeding disrupts microbial diversity, reduces beneficial taxa, increases proinflammatory species, impairs intestinal barrier function, and promotes metabolic endotoxemia and systemic inflammation [20]. In this study, 16S rRNA sequencing revealed that BTF profoundly reshaped the gut community structure.

BTF increased the relative abundance of Lactobacillus, Allobaculum, and Muribaculaceae, which are widely associated with improved metabolic health, reduced inflammation, and enhanced intestinal barrier function [21,22]. Lactobacillus strains are known to improve lipid profiles, reduce foam cell formation, and attenuate atherosclerotic lesions. Muribaculaceae is negatively correlated with obesity and metabolic dysfunction [23]. Meanwhile, BTF reduced the abundance of proatherogenic and proinflammatory taxa. LEfSe and LDA analysis further confirmed that BTF enriched specific microbial signatures associated with cardiovascular protection.

These results reveal a previously unreported mechanism: BTF attenuates atherosclerosis partly by restoring gut microbiota homeostasis. This microbiota-mediated effect likely acts synergistically with direct hepatic lipid regulation to achieve comprehensive protection. This study establishes a previously unrecognized link between the anti-atherosclerotic effects of BTF and gut microbiota modulation, offering new insights into its multi-target mechanism.

### 4.7. Limitations of the Present Study

Several limitations should be acknowledged. First, this study was conducted in ApoE^−/−^ mice, which cannot fully recapitulate human atherosclerosis. Second, although we identified seven absorbed flavonoids, their individual contributions and synergistic interactions require further investigation using purified compounds. Third, we did not explore the upstream signaling regulating *SREBP1*, such as the *AMPK*, *PI3K*/*Akt*, or Insig/*SCAP* pathways. Fourth, the functional metabolites of gut microbiota, such as short-chain fatty acids, bile acids, and trimethylamine N-oxide, were not measured. Fifth, we did not explore the anti-inflammatory effects of BTF in detail, including macrophage polarization and inflammatory cytokine profiles.

### 4.8. Future Directions

Based on the present findings, we propose several future research directions to advance clinical translation: First, in vitro mechanistic studies will be performed to clarify how key BTF flavonoids regulate *SREBP1* cleavage, nuclear translocation, and transcriptional activity, including upstream kinases such as *AMPK* and *PI3K*/*Akt* [24,25]. Second, microbiota depletion and fecal microbiota transplantation will be used to confirm the causal role of gut microbiota in BTF’s effects, rather than just an association [26,27]. Third, targeted metabolomics will be applied to analyze changes in SCFAs, bile acids, and other microbial metabolites that mediate host–microbe interactions [28,29]. Fourth, long-term toxicity and pharmacokinetic studies will be conducted to support safety profiles for potential clinical use [30,31]. Fifth, future studies will explore the anti-inflammatory effects of BTF, focusing on *NF-κB* signaling, macrophage foam cell formation, and vascular inflammation [32,33].

Ultimately, we aim to develop BTF into a standardized, quality-controlled nutraceutical or phytopharmaceutical for primary prevention and adjunctive treatment of atherosclerosis and related cardiovascular diseases.

## 5. Conclusions

In conclusion, total flavonoids from *Berberis kaschgarica* Rupr. (BTF) significantly attenuate high-fat diet-induced atherosclerosis in ApoE^−/−^ mice. BTF acts through a coordinated multi-target mechanism: (1) inhibiting hepatic *SREBP1*-dependent lipogenesis and VLDL assembly to improve dyslipidemia; (2) enhancing systemic antioxidant capacity to reduce oxidative vascular injury; and (3) restoring gut microbiota homeostasis to reduce metabolic inflammation. This study provides novel mechanistic insights and preclinical evidence supporting BTF as a promising natural candidate for the prevention and treatment of atherosclerosis.

## Figures and Tables

**Table 1:** The identified compounds by UPLC-MS/MS.

Compounds	Formula	Retention Time (min)	ION Mode	Adducts	*m*/*z*
Kaempferol	C_15_H_10_O_6_	22.12	NEG	M-H	285.0421
Diosmetin	C_16_H_12_O_6_	22.34	NEG	M-H	299.0565
Apigenin	C_15_H_10_O_5_	21.96	POS	M+H	271.0614
Acacetin	C_16_H_12_O_5_	26.32	POS	M+H	285.0730
Fortunellin	C_28_H_32_O_14_	20.89	NEG	M+FA-H	268.037
Dihydromyricetin	C_15_H_12_O_8_	13.38	NEG	M-H	319.0466
Calycosin	C_16_H_12_O_5_	21.03	POS	M+NH4	302.1015

## Data Availability

The datasets (GSE10092, GPL7077) for this study can be found in the GEO database [https://www.ncbi.nlm.nih.gov/geo/ (accessed on 10 March 2025)], SIB Swiss Institute of Bioinformatics (SwissTargetPrediction) [http://www.swisstargetprediction.ch/ (accessed on 20 January 2026)], GeneCards Database [https://www.genecards.org/ (accessed on 30 March 2026)], the OMIM Database [https://omim.org/ (accessed on 10 March 2026)], DrugBank Database [https://www.drugbank.com/ (accessed on 25 March 2026)], and STRING database [https://cn.string-db.org/ (accessed on 12 February 2026)].

## Abbreviations

The following abbreviations are used in this manuscript:

AS	Atherosclerosis
BTF	Total flavonoids from *Berberis kaschgarica* Rupr.
TC	Total cholesterol
TG	Triglycerides
LDL-C	LDL cholesterol
HDL-C	HDL cholesterol
MDA	Malondialdehyde
SOD	Superoxide dismutase
GSH	Glutathione
CAT	Catalase
LDH	Lactate dehydrogenase
